# Ultrathin 2D Metal–Organic Framework Nanosheets In situ Interpenetrated by Functional CNTs for Hybrid Energy Storage Device

**DOI:** 10.1007/s40820-020-0382-x

**Published:** 2020-02-04

**Authors:** Feitian Ran, Xueqing Xu, Duo Pan, Yuyan Liu, Yongping Bai, Lu Shao

**Affiliations:** 1 MIIT Key Laboratory of Critical Materials Technology for New Energy Conversion and Storage, State Key Laboratory of Urban Water Resource and Environment (SKLUWRE), School of Chemistry and Chemical Engineering, Harbin Institute of Technology, Harbin, 150001 People’s Republic of China; 2 Key Laboratory of Materials Processing and Mold (Zhengzhou University), Ministry of Education, National Engineering Research Center for Advanced Polymer Processing Technology, Zhengzhou University, Zhengzhou, 450002 People’s Republic of China

**Keywords:** Metal–organic frameworks, Carbon nanotubes, Ultrathin 2D nanosheets, Hybrid supercapacitor

## Abstract

- The ultrathin nickel metal–organic framework (MOF) nanosheets in situ interpenetrated by functional carboxylated carbon nanotubes (C-CNTs) were successfully constructed. The incorporated C-CNTs effectively adjust the layer thickness of Ni-MOF nanosheets.
- The integrated hybrid MOF nanosheets delivered the boosted electrochemical performances and exhibited superior specific capacity of 680 C g^−1^ at 1 A g^−1^.

**Electronic supplementary material:**

The online version of this article (10.1007/s40820-020-0382-x) contains supplementary material, which is available to authorized users.

## Abstract (article highlights)

The controllable construction of two-dimensional (2D) metal–organic framework (MOF) nanosheets with favorable electrochemical performances is greatly challenging for energy storage. Here, we design an in situ induced growth strategy to construct the ultrathin carboxylated carbon nanotubes (C-CNTs) interpenetrated nickel MOF (Ni-MOF/C-CNTs) nanosheets. The deliberate thickness and specific surface area of novel 2D hybrid nanosheets can be effectively tuned via finely controlling C-CNTs involvement. Due to the unique microstructure, the integrated 2D hybrid nanosheets are endowed with plentiful electroactive sites to promote the electrochemical performances greatly. The prepared Ni-MOF/C-CNTs nanosheets exhibit superior specific capacity of 680 C g^−1^ at 1 A g^−1^ and good capacity retention. The assembled hybrid device demonstrated the maximum energy density of 44.4 Wh kg^−1^ at a power density of 440 W kg^−1^. Our novel strategy to construct ultrathin 2D MOF with unique properties can be extended to synthesize various MOF-based functional materials for diverse applications.

**Electronic supplementary material:**

The online version of this article (10.1007/s40820-020-0382-x) contains supplementary material, which is available to authorized users.

## Graphical abstract

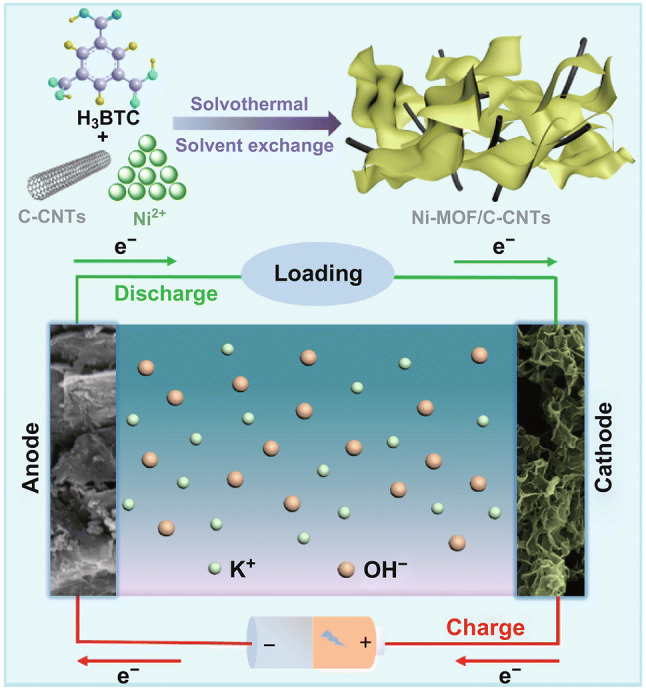

## Introduction

The aggravations of environmental pollution and global warming have deteriorated the human living conditions and sustainable development of modern economy [1, 2]. Being confronted these issues, the “green” strategies by exploiting the storage technologies of sustainable and renewable energy sources have become an international consensus [3, 4]. Therein, the electrochemical energy storage systems (EESs) are being accredited as one of the most potential devices for efficient energy storage [5–7]. As the typical representative, supercapacitors (SCs) have widely aroused scientific and technological interests due to their high-power output, fast charge–discharge kinetics, and long cycle life [8–10]. These advantages make them become the favorable candidates for applications of portable electronic device or hybrid electric vehicles [11, 12]. Nonetheless, inferior energy density of SCs has hindered their extensive application in the near future. To finely overcome this drawback, numerous efforts have been contributed to develop novel technical routes [1, 13, 14]. A promising strategy is the fabrication of hybrid supercapacitors (HSCs), which not only could deliver high energy density by virtue of the reversible faradaic reaction occurred at positive electrodes, but also maintain the high-power yield of capacitive-type negative electrodes [15, 16]. Therefore, the rational design of HSCs with integrated advantages of battery-type and capacitive electrodes will be an applicable route to realize high energy density without sacrificing the specific power.

In general, the energy density of HSCs mainly depends on the electrochemical performances of battery-type electrodes. Thus, considerable efforts have been channeled into designing and exploiting the high-performance electrode materials, especially in the transition metal compounds [17–19]. Emerging as a new category of crystalline porous materials with ultrahigh porosity and abundant metal active sites, metal–organic frameworks (MOFs) are assembled by the coordination bond between metal ions and organic ligands, giving the enormous application prospects [20–22]. Recently, extensive researches related to transition metal-based MOFs have been demonstrated that they are the appealing candidates for energy conversion and storage [23–25]. However, most MOFs electrodes unavoidably suffer from inherently low electrical conductivity and instability, which has greatly thwarted their capacitive performances and rate capacities [26, 27]. Up to now, numerous approaches have focused on the construction of novel topologies and tuning of orientation, morphology, and constituent to compensate the intrinsic defects of MOFs. For instance, Liu et al. [28] synthesized kinetically stable nickel-based pillared MOFs via ligand functionalization. The as-synthesized Ni-DMOF-ADC electrode exhibited desired specific capacitance of 552 F g^−1^ at 1 A g^−1^ and excellent cycling stability. Zheng et al. [23] employed Co(OH)_2_ as template to guide MOF orientation and convert itself into MOF construction. As a result, the uniformly vertical orientation of CoNi-MOF ensures the sufficient redox active sites and short ion transport pathways, thus achieving excellent electrochemical performances. Besides, atom doping, mechanical exfoliation, and acid regulation have also been developed to tune the microstructure, crystalline, and electronic structures of MOF [29–31]. Another strategy, the exploitation of conductive MOFs seems an attractive approach, but the synthesis cost and ligand screening delay their research advances [27, 32]. Then, to develop new-style conductive MOFs may be the long-term perspective. Withstanding the aforementioned advances so far, the poor electron transfer resulting from discrete MOFs nanoparticles still inevitably exists. What is more, the accessible active sites of electrolyte ion were also enslaved to the microstructure, thus offering imperfect capacitive performance. Therefore, how to construct the continuous MOFs network microstructure for efficient electron transfer as well as improved ion diffusion, and thus boosting electrochemical reaction kinetics, is highly challenged.

Recently, 2D MOFs have aroused tremendous attention in investigation of environment and energy fields owing to their thickness on the atomic scale and inherent characteristic that differing from those of bulk lamellar structures [33, 34]. Particularly, confining the thickness of intrinsically non-layered structure into nanoscale and keeping a large lateral dimension are likely to change their inter-particle connectivity [12, 35]. For ultrathin nanosheets, moreover, the active sites can be sufficiently exposed, which is capable of tuning the band gap energy and the density of states near the Fermi level, thus enhancing electrical conductivity [36–39]. The exposed metal active sites could serve as highly active centers to decrease the energy barrier of faradaic redox reactions and to facilitate the capture ability of electrolyte ions, which is conducive to improve the electrochemical reaction kinetics [35, 38, 40]. Stimulated by these superiorities, the controllable construction of ultrathin MOFs nanosheets for energy storage is a promising research direction but still remains a challenge.

Herein, we demonstrate an in situ induced growth strategy to interpenetrate carboxylated carbon nanotubes (C-CNTs) into the ultrathin 2D nickel MOF (Ni-MOF/C-CNTs) nanosheets. Owing to the unique anisotropic properties, one-dimensional (1D) C-CNTs not only could provide fast axial electron transport and short ion diffusion pathways, but also effectively adjust the layer thickness of Ni-MOF and buffer the volume change. Benefitting from its distinct structure, the integrated hybrid MOFs nanosheets were endowed with abundant electroactive sites, thus considerably boosting the electrochemical performances. As a demonstration, the Ni-MOF/C-CNTs40 exhibits superior specific capacity and desired rate performances. Besides, the assembled hybrid device based on Ni-MOF/C-CNTs40 demonstrated potential toward the application of supercapacitor.

## Experimental Section

### Synthesis of Ni-MOF and Ni-MOF/C-CNTs Nanosheets

Typically, 40 mg C-CNTs were dispersed in the 60 mL DMF containing 0.5 mmol H_3_BTC with the assistance of ultra-sonication, and then, Ni(NO_3_)_2_·6H_2_O (3 mmol) was poured into the above suspension. After ultrasonic dissolving for 60 min, the obtained precursor suspension was transferred into a 100 mL autoclave and kept at 150 °C for 12 h before it was cooled naturally. The resultant product was soaked in methanol for 48 h to remove solvents and unreacted ligand. Finally, the precipitates were centrifuged, washed with ethanol, and then dried in vacuum at 80 °C for 24 h. The resulting products were named as Ni-MOF/C-CNTsX (*X* = 20, 40, 60), where x represent various amounts of C-CNTs. For comparison, pure Ni-MOF and MOF/CNTs40 (containing 40 mg CNTs) were also synthesized as given in the above procedure. The production yield and electronic photographs of appearance for Ni-MOF and Ni-MOF/C-CNTs40 are also provided in Table S1 and Fig. S1.

### Electrochemical Measurements and Analysis

Electrochemical measurements were conducted on a CHI 660E electrochemical workstation (CH, Instruments, Inc., Shanghai). In three-electrode system, the 3 M KOH aqueous solution was used as electrolyte, and the Pt foil and Hg/HgO electrode were performed as counter electrode and reference electrode, respectively. To prepare working electrode, 80 wt% Ni-MOF, Ni-MOF/C-CNTs, or Ni-MOF/CNTs40 as active materials, 10 wt% acetylene black, and 10 wt% binder (polyvinylidene fluoride, PVDF) were mixed with moderate NMP to form a homogeneous slurry and then coated onto the cleaned Ni foams. Subsequently, the Ni foam electrode was completely dried at 80 °C for 24 h and pressed at 10 MPa to obtain the working electrode. The mass loading of the active material in each working electrode was around 2 mg cm^−2^. The cyclic voltammograms (CV) were performed in the potential range of 0 ~ 0.7 V at various scan rates. The charge–discharge tests were operated between 0 and 0.54 V at various current densities. The electrochemical impedance spectroscopy (EIS) was conducted at frequency range of 100 kHz to 0.01 Hz.

### Assembling of HSC Device

To assemble the HSC device, 80 wt% commercial active carbon (AC), 10 wt% acetylene black, and 10 wt% PVDF were mixed uniformly with NMP and then coated onto cleaned nickel foam to obtain negative electrode. The positive electrode was prepared using Ni-MOF/C-CNTs40 as active materials via the same process. The mass loading of positive and negative electrodes was calculated by the principle of charge balance (see Formula S3). The HSC device was assembled using NKK separator to separate positive and negative electrodes and 3 M KOH electrolyte in a split test cell (MTI Corporation). The cycle stability of HSC device was evaluated by GCD technique on a LAND-BTS battery test system.

The relevant calculation formulas are all provided in the Supporting Information.

## Results and Discussion

**Fig. 1:**
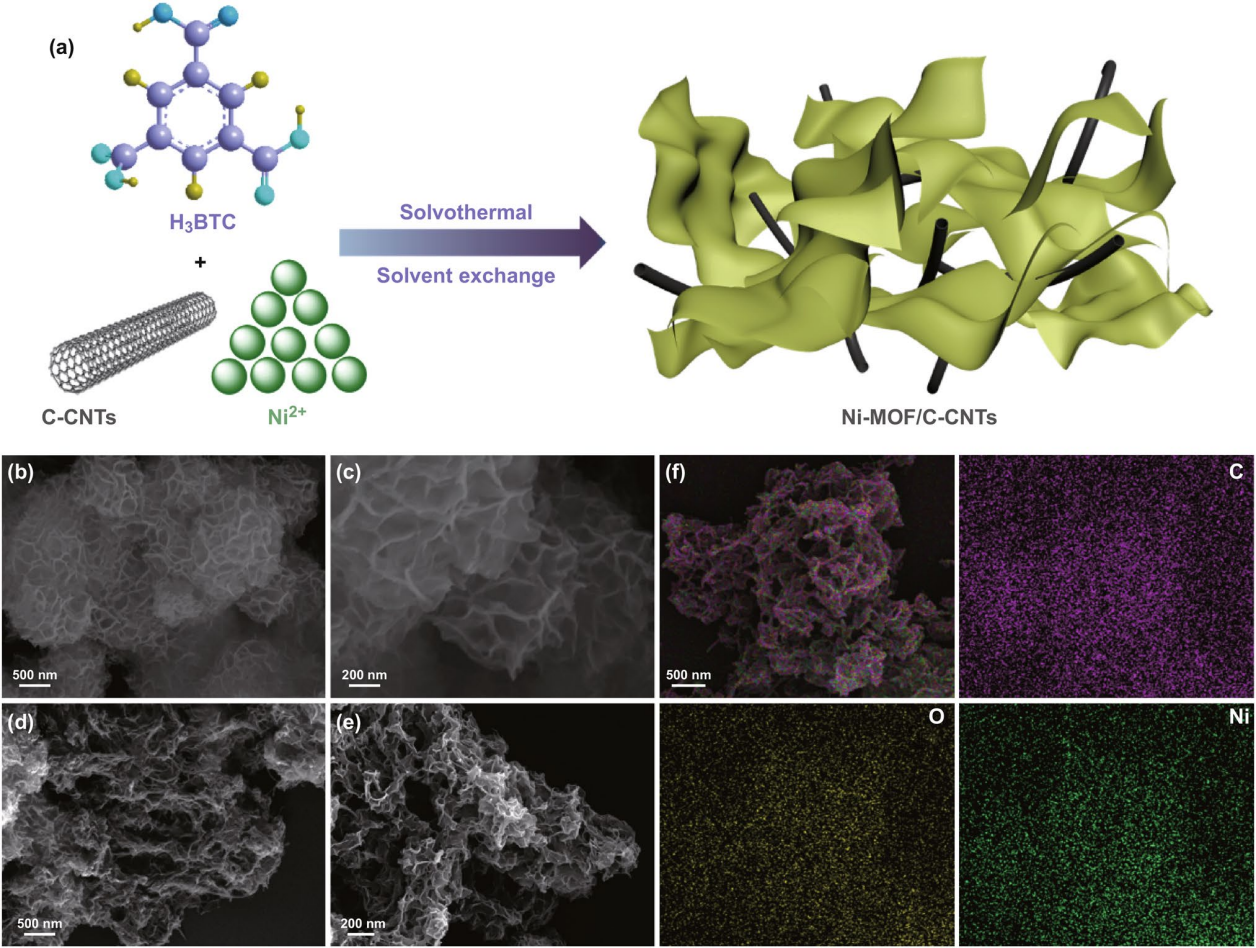
a Schematic illustration for the synthesis of ultrathin Ni-MOF/C-CNTs nanosheets. SEM images of as-synthesized **b**, **c** Ni-MOF, and **d**, **e** Ni-MOF/C-CNTs40 nanosheets. **f** EDS mapping for C, O, and Ni elements of the Ni-MOF/C-CNTs40

The assembly procedure of 2D well-interconnected Ni-MOF/C-CNTs ultrathin nanosheets is schematically shown in Fig. 1a, which mainly involved solvothermal reaction and solvent exchange steps. Scanning electron microscopy (SEM) images were employed to distinguish the morphological evolution. In the absence of C-CNTs, Ni-MOF would freely nucleate and grow, thus resulting in layer-by-layer stacking structures comprised of aggregated 2D nanosheets with a lateral size of 1–2 μm, as observed in Fig. 1b, c. When moderate amount of C-CNTs (Fig. S2a, b) was introduced reaction system, noticeably, the ultrathin and highly wrinkled Ni-MOF nanosheets are formed (Fig. 1d, e), revealing the distinctive role of the C-CNTs. Moreover, in sharp contrast with the pure Ni-MOF, the typical Ni-MOF/C-CNTs40 generated large amount of highly interconnected loose and wrinkled nanostructure constructed of thinner 2D nanosheets. In this step, attributed to the C/O functional groups (−COOH, −C–OH) of C-CNTs, the free Ni^2+^ could be absorbed on the surface of C-CNTs via electrostatic attraction, thus forming nucleation centers to guide the crystal and growth of Ni-MOF [38]. After deprotonated H_3_BTC ligands coordinated to Ni^2+^ ions, the bridged ordered chain has self-assembled into 2D Ni-MOF nanosheets along with C-CNTs skeleton. In this design, the moderate C-CNTs could effectively adjust aggregation degree of Ni-MOF, thus forming loose sheet-like structure. Such 2D well-interconnected nanosheets can provide abundant ion accessible space and electroactive sites, thus enabling fast ion diffusion/transportation and enhanced reaction kinetics. Further energy-dispersive spectroscopy (EDS) elemental mapping of Ni-MOF/C-CNTs40 (Fig. 1f) showed the homogeneous distributions of Ni, O, C elements, revealing C-CNTs have been interpenetrated into Ni-MOF framework and formed homogeneous nanostructure. Besides, the influence of C-CNTs amount and CNTs on microstructure evolution of Ni-MOF was also systematically investigated, and the corresponding surface morphologies were elucidated by SEM images. We observe that the small amount of C-CNTs has indistinctive influence on Ni-MOF morphology evolution, and stacked nanosheets still exist (Fig. S3a, b); nevertheless, with the content of C-CNTs in Ni-MOF/C-CNTs increasing continuously, an agglomeration tendency could be observed (Fig. S3c, d), which may be ascribed to the sharp increase in nucleation sites. These results indicate that the appropriate amount of C-CNTs plays a key role in the construction of thin 2D well-interconnected nanosheets. However, regarding Ni-MOF/CNTs40, there appears loose morphology that is different with layer-by-layer stacking structures of Ni-MOF, suggesting CNTs has the effect on crystallization of Ni-MOF. However, we can observe plentiful CNTs on the surface of Ni-MOF/CNTs40 hybrid instead of embedding in Ni-MOF (Fig. S4), which is attributed to the absence of the C/O groups on CNTs and thus cannot provide nucleate site for growth of Ni-MOF.

**Fig. 2:**
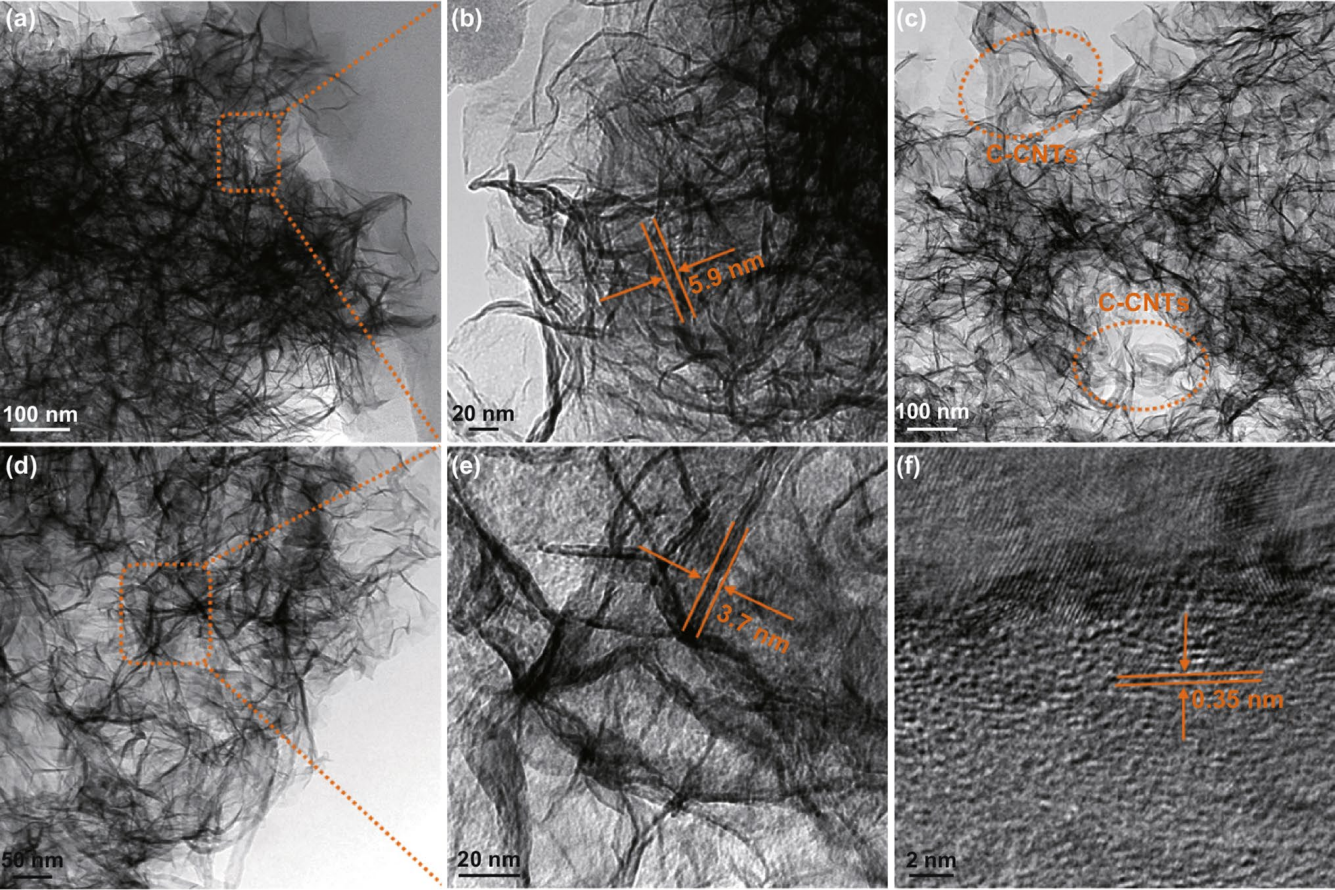
TEM images of **a**, **b** Ni-MOF and **c–e** Ni-MOF/C-CNTs40 nanosheets with different magnification. **f** HRTEM image of Ni-MOF/C-CNTs40

The ultrathin nature of Ni-MOF and Ni-MOF/C-CNTs40 was further discerned by transmission electron microscopy (TEM). As explicated in Fig. 2a, b, the pure Ni-MOF presents noticeable nanosheet structure, and the thickness of nanosheets is about 5–8 nm. As for Ni-MOF/C-CNTs40, it can be obviously observed that C-CNTs were homogeneously embedded in the Ni-MOF nanosheets, and sheet-like nanostructure extended along the C-CNTs backbone, as shown in Fig. 2c, d. Moreover, Ni-MOF/C-CNTs40 reveals highly porous nanostructure and a sheet thickness of about 3–4 nm (Fig. 2e), which is thinner than that of pure Ni-MOF, giving convictive evidence for unique functionality of C-CNTs. In high-resolution TEM (HRTEM) image (Fig. 2f), several different lattice fringes could be obviously detected, wherein the lattice spacing of 0.35 is consistent with the (002) plane of C-CNTs. These results confirm that the incorporated C-CNTs provide the backbone for Ni-MOF crystal growth, thus generating a 2D sheet-like hybrid nanostructure.

**Fig. 3:**
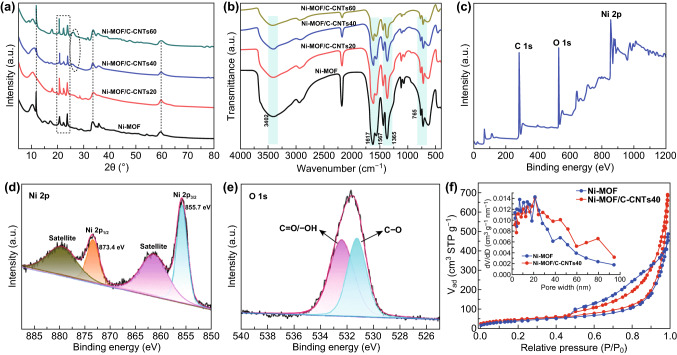
Physical characterization of as-synthesized samples. **a** XRD patterns and **b** FT-IR spectra of Ni-MOF and a series of Ni-MOF/C-CNTs nanostructures. **c** XPS spectrum of Ni-MOF/C-CNTs40. High-resolution XPS spectra of **d** Ni 2p and **e** O 1 s. **f** N_2_ adsorption–desorption isotherms and pore size distribution plots (inset) of Ni-MOF and Ni-MOF/C-CNTs40

The crystal structure of Ni-MOF and Ni-MOF/C-CNTs was characterized via powder X-ray diffraction (XRD) patterns (Fig. 3a). The intense diffraction peaks at about 10^o^ and 22^o^ can correspond to (100) and (101) planes, respectively, which are typical diffraction peaks of Ni-MOF coordinated by Ni^2+^ and H_3_BTC and well matching with previous reports [41, 42]. After introducing C-CNTs, all samples exhibit similar phase structure expect some diffraction peaks shifted toward lower angle caused by the slight change of layer distance, indicating the incorporation of C-CNTs has no alteration for the crystallization of Ni-MOF. Moreover, with the additive amount of C-CNTs increasing, the diffraction peaks appeared at 26^o^ can be assigned to (002) plane of graphitic structure of carbon, which are well consistent with TEM result, further demonstrating the presence of C-CNTs in Ni-MOF/C-CNTs [43–45]. Besides, the chemical species of Ni-MOF and Ni-MOF/C-CNTs was verified by FT-IR spectra. As revealed in Fig. 3b, the broad and intense absorption peaks at about 3402 cm^−1^ correspond to the O–H stretching vibration, representing the existence of coordinated H_2_O molecules within the Ni-MOF interlayers [41, 46]. The characteristic peaks for asymmetric stretching modes of coordinated COO^−^ groups and aromatic C=C groups of BTC ligands appear at 1617 and 1567 cm^−1^, respectively [47]. Also, the symmetrical stretching vibrations of COO^−^ could be observed at 1365 cm^−1^ [48]. The separation for two COO^−^ stretching modes shows that H_3_BTC has coordinated with Ni^2+^ and formed polymeric structure. Besides, the characteristic peaks at 765 cm^−1^ could be ascribed to the plane vibration of substituted benzene core in trimesic acid [47]. The integration of Ni-MOF and C-CNTs was further identified via Raman spectroscopy. As revealed in Fig. S5, two characteristic bands at 1461 and 1596 cm^−1^ are associated with the vibration stretching of benzene ring and a complex stretching mode of carboxylate group, respectively, which are similar to previous reports, further indicating the successful formation of Ni-MOF [42, 43, 49]. After introducing C-CNTs, two bands appeared at 1353 and 1585 cm^−1^, which are indexed to D and G bands, representing the disordered and graphite structure of C-CNTs [43]. Also, another band appeared at 2703 cm^−1^ is assigned to 2D peak of C-CNTs. In the spectra of Ni-MOF/C-CNTs, there exist no obvious peaks of Ni-MOF, which may be caused by the strong peaks of C-CNTs.

The surface chemical composition and valence states of typical Ni-MOF/C-CNTs40 were estimated by the XPS technique. As shown in Fig. 3c, the XPS survey spectrum clearly manifests the presence of C, O, and Ni elements, which match well with the aforementioned EDS results. In high-resolution Ni 2p spectra, as shown in Fig. 3d, two main peaks at about 855.7 and 873.4 eV are assigned to Ni 2p_3/2_ and Ni 2p_1/2_ spin orbits, respectively [50, 51]; as well, two distinct peaks centered at around 861.6 and 880.1 eV are shake-up satellites of Ni 2p_3/2_ and Ni 2p_1/2_, respectively, which are in accordance with that of previous reports, demonstrating that the existence form of nickel ions in the Ni-MOF/C-CNTs nanosheets is divalent state [43, 48]. Regarding the O 1 s spectrum, as revealed in Fig. 3e, it can be well fitted with two peaks at around 531.2 and 532.4 eV, which can be attributed to oxygen in C=O/O–H groups and C–O group, respectively. Such result should be associated with the carboxylate group and hydroxide group in the trimesic acid ligand and C-CNTs. As for C 1 s spectrum (Fig. S6), two major peaks at 284.5 and 288.5 eV correspond to phenyl carbons (C=C) and carboxylate carbons (O–C=O), respectively [50, 52, 53].

N_2_ adsorption–desorption measurements were employed to analyze the specific surface area and porous nature of as-synthesized samples. As shown in Fig. 3f, the N_2_ adsorption–desorption isotherms of Ni-MOF and Ni-MOF/C-CNTs40 illustrate typical type IV isotherm with a clear hysteresis loop of typical H2, verifying their mesoporous characteristic. The BET specific surface area (S_BET_) of Ni-MOF and Ni-MOF/C-CNTs40 is 140.3 and 155.7 m^2^ g^−1^, and the corresponding total pore volume is 0.74 and 1.05 cm^3^ g^−1^, respectively, which indicate that the introduction of C-CNTs has improved the microstructure, thus boosting its S_BET_ and pore volume. The resultant pore size distributions of Ni-MOF and Ni-MOF/C-CNTs40 are shown in Fig. 3f. As presented, mesoporous structure is obviously acquired for samples. In contrast to the average pore diameter of 21 nm for Ni-MOF, that of Ni-MOF/C-CNTs40 increases to 26.9 nm. These mesoporous structures can significantly boost the utilization efficiency of the electrochemical active sites because it can accelerate mass transfer; moreover, the higher specific surface area could provide enough contract area for electrode and electrolyte ions [54].

**Fig. 4:**
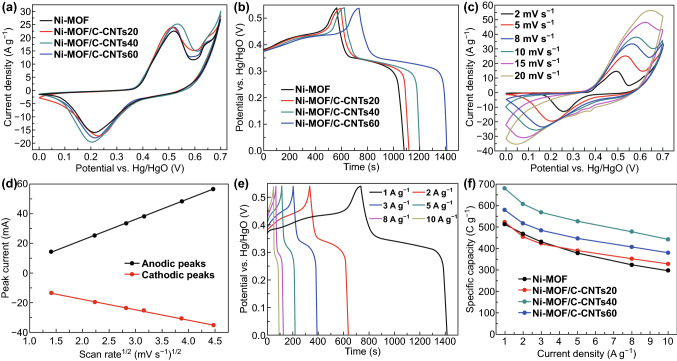
Electrochemical evaluation measured in 3 M KOH electrolyte with a three-electrode system. **a** CV curves at 10 mV s^−1^ and **b** GCD curves at 1 A g^−1^ for Ni-MOF and a series of Ni-MOF/C-CNTs nanostructures. **c** CV curves at different scan rates, **d** the relationship between the scan rate and peak current density, and **e** GCD curves at various current densities for Ni-MOF/C-CNTs40. **f** Specific capacities for Ni-MOF and a series of Ni-MOF/C-CNTs

Encouraged by their unique 2D structure, the as-synthesized Ni-MOF and Ni-MOF/C-CNTs hybrid was directly used as active components to fabricate electrodes. The electrochemical performances were systematically investigated in the three-electrode system by using 3 M KOH as an aqueous electrolyte. The CV curves of Ni-MOF and a series of Ni-MOF/C-CNTs hybrids are displayed in Fig. 4a. A pair of redox peaks along with the clear potential separations could be identified in all CV curves, which is an indicative feature of battery-type electrode. This typical characteristic is generally considered as the reversible transformation between Ni(II) and Ni(III) on account of faradaic redox reaction under the presence of OH^−^, which can be described as Eqs. 1 and 2 [48, 55, 56]:

1 $${\text{Ni}}\left( {\text{II}} \right) \, + {\text{ OH}}^{ - } \leftrightarrow {\text{Ni}}\left( {\text{II}} \right)\left( {\text{OH}} \right) \, + {\text{ e}}^{ - }$$

2 $${\text{Ni}}\left( {\text{II}} \right)\left( {\text{OH}} \right) \leftrightarrow {\text{Ni}}\left( {\text{III}} \right)\left( {\text{OH}} \right) \, + {\text{ e}}^{ - }$$

Evidently, the integral area of CV curves for Ni-MOF/C-CNTs was larger than that for Ni-MOF electrode, indicative of higher capacity of charge storage. The similar tendency can also be observed in galvanostatic charge–discharge (GCD) curves (Fig. 4b). Compared to Ni-MOF, the longer discharge time demonstrates the higher specific capacity of Ni-MOF/C-CNTs, wherein the longest discharge time of Ni-MOF/C-CNTs40 reveals its optimal charge storage capacity. Also, the nonlinear behavior and distinct potential plateau of GCD curves are well matched with the CV results, further illustrating their faradaic reaction processes. Such increment for Ni-MOF/C-CNTs40 can be attributed to the fact that well-interpenetrated C-CNTs effectively adjusted the layer thickness of Ni-MOF and enhanced the electrical conductivity of integrated nanoarchitecture, as well as increased the specific surface area, thus enabling the fast axial electron transport, short ion diffusion pathways, and enough accessibility of electroactive sites to the electrolyte ions. To assess the rate capability of the typical Ni-MOF/C-CNTs40 electrode, the CV and GCD tests were performed. As observed in Fig. 4c, with the scan rate of CV curves increasing, the separation between anodic and cathodic current peaks increased, which is possibly related with intrinsic resistance [57]. But it should be noted that the symmetrical redox peaks even at high scan rates revealed the good reversibility of faradaic reactions for the obtained Ni-MOF/C-CNTs40 hybrid nanoarchitecture. Furthermore, the cathodic and anodic peak current (*I*_p_) presented a well linear correlation with the square root of scan rate (*ν*^*1/2*^), suggesting the electrode kinetics of MOF/C-CNTs40 is determined by diffusion-controlled process (Fig. 4d) [58, 59].

The GCD behavior of Ni-MOF/C-CNTs40 was further employed to study its charge storage performance (Fig. 4e). The clear voltage plateau regions elucidate the battery-type characteristic of Ni-MOF/C-CNTs40 electrode, which correspond to the redox process of CV results. Based on discharge time, the specific capacities of Ni-MOF/C-CNTs40 were calculated as 680, 606, 569, 527, 478, and 442 C g^−1^ at 1, 2, 3, 5, 8, and 10 A g^−1^, respectively. Such values exceed that of some previously reported MOF-based electrode materials (Table S2). To further have insight into the effect of incorporated C-CNTs on the charge storage capacity, detailed GCD tests of Ni-MOF, Ni-MOF/C-CNTs20, Ni-MOF/C-CNTs60, and Ni-MOF/CNTs40 were examined. As shown in Fig. S7, all GCD curves exhibit similar profile, indicating the same redox mechanisms in the charging–discharging process. The corresponding specific capacities as the function of current densities are shown in Fig. 4f. In details, the specific capacities of Ni-MOF, Ni-MOF/C-CNTs20, Ni-MOF/C-CNTs40, and Ni-MOF/C-CNTs60 are 517, 524, 680, and 581 C g^−1^ at 1 A g^−1^, and the specific capacity retentions of them are 57.3%, 62.4%, 65%, and 65.6% from 1 to 10 A g^−1^, respectively. As for Ni-MOF/CNTs40, its specific capacity is 540 C g^−1^ at 1 A g^−1^ and capacity retention is 60.5% from 1 to 10 A g^−1^ (Fig. S8). Evidently, owing to the boosted charge transfer kinetics of the redox reaction and ion diffusion/migration by incorporated moderate C-CNTs, the Ni-MOF/C-CNTs hybrids delivered superior performance in terms of much higher specific capacities and rate capability compared with pure Ni-MOF. Nevertheless, the excess C-CNTs enabled serious agglomeration and low percentage of exposed active surfaces, which greatly restrict the diffusion of electrolyte and reaction kinetics, thus resulting in the inferior performances. In addition, the introduced CNTs only have a slight contribution to the specific capacity and rate capability of Ni-MOF, which may be ascribed to the fact that formed loose structure is not well interconnected and thus cannot provide fast axial electron transport.

In order to further assess the charge transfer characteristics, the electrochemical impedance spectra (EIS) were conducted. As shown in Fig. S9, the Nyquist plots of Ni-MOF and Ni-MOF/C-CNTs40 are all consisted of a semicircle and a straight line. Normally, the intercepts on real axis represent equivalent series resistances (*R*_s_), while the diameter of semicircle represents charge transfer resistance (*R*_ct_), and the straight slope corresponds to the Warburg impedance (*Z*_w_*)* related to the electrolyte ion diffusion in electrode [57, 60, 61]. The *R*_s_ values of Ni-MOF and Ni-MOF/C-CNTs40 are 1.02 and 0.99 Ω, respectively, indicating their small equivalent series resistances. Moreover, obviously reduced diameter of the semicircle and steeper line slope were obtained for Ni-MOF/C-CNTs40, signifying its enhanced charge transfer kinetics of the redox reaction and faster ion diffusion/migration in the electrolyte, which indicate the significant role of C-CNTs working as a highway for electron/ion transport, as well as improving the electrical conductivity and facilitating electrochemical reaction kinetics.

**Fig. 5:**
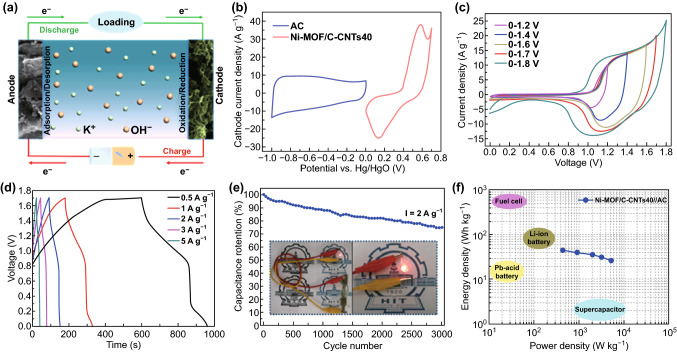
a Schematic illustration of the MOF/C-CNTs40//AC HSC device. **b** CV curves of Ni-MOF/C-CNTs40 and commercial AC electrodes at 5 mV s^−1^ in three-electrode system, respectively. **c** CV curves of the HSC device in various voltages at 20 mV s^−1^. **d** GCD curves of the HSC device at various current densities. **e** Cycling performance at the current density of 2 A g^−1^ and two devices in series can lighten up a red LED indicator (inset). **f** Ragone plots of HSC device

To verify the potential applications of as-synthesized nanoarchitecture, a hybrid supercapacitor (HSC) was assembled by utilizing Ni-MOF/C-CNTs40 as battery-type electrode and commercial active carbon (AC) as capacitive-type electrode (Fig. 5a). The HSC integrates the charge storage characteristics of battery-type behavior originated from reversible faradaic redox reaction and capacitive behavior correlated well with cations adsorption/desorption process, which is expected to broaden operating voltage window, thus delivering boosted energy density [62, 63]. The appropriate voltage window of HSC was estimated by the CV curves of Ni-MOF/C-CNTs40 and AC in three-electrode system. As presented in Fig. 5b, obviously, the Ni-MOF/C-CNTs40 and commercial AC can well work in the potential range of 0–0.7 V and − 1–0 V, respectively. To better optimize the voltage window of assembled HSC, a series of CV tests at different voltage windows ranging from 1.0 to 1.8 V were conducted (Fig. 5c). Evidently, it cannot be observed any noticeable distortion of the CV curve within 1.7 V; however, with further expansion of operating voltage to be 1.8 V, an obvious polarization phenomenon associated with oxygen evolution reaction revealed electrolyte decomposition/water splitting [64]. Thus, the voltage window was optimized to be 1.7 V. Figure S10 shows the CV curves of HSC device at 5 to 75 mV s^−1^. The better CV shape preservation at higher scan rate implies good rate performance. Moreover, the GCD curves at various current densities (Fig. 5d) also illuminate that the HSC can be well operated within an ideal voltage window of 0–1.7 V. Based on the discharged time, the specific capacitance of the Ni-MOF/C-CNTs40//AC HSC device can be calculated and is shown in Fig. S11. The HSC device delivers a maximum specific capacitance of 97.6 F g^−1^ at 1 A g^−1^ and a capacitance retention of 66.7% at 5 A g^−1^, indicating its desired charge storage ability. Besides, the cycling performance of HSC device was assessed by continuous charge–discharge test in the voltage window of 0–1.7 V at 2 A g^−1^. After 3000 sequential charging–discharging cycles (Fig. 5e), the HSC device exhibits 77% retention of the initial specific capacitance. According to previous reports, such capacitance decay was mainly attributed to the partly irreversible phase transition that formed in the redox process. Hence, developing phase engineering to optimize the crystal structures and thus decreasing the structure change occured in the charge/discharge process are very significant for specific need of MOF-based electrodes in future study. To verify its practical application, two assembled HSCs connected in series were used to light a red LED (inset of Fig. 5e) after being charged to 3.4 V, indicating the high-power output of Ni-MOF/C-CNTs40//AC device.

Energy density (*E*) and power density (*P*) are essential evaluation indexes for energy storage devices. In view of wide voltage of 1.7 V and desired specific capacitance, it is greatly expected to acquire excellent energy density for Ni-MOF/C-CNTs40//AC HSC. As displayed in Fig. 5f, the Ragone plot in respect of energy density and power density of the HSC was calculated from discharge curves. The HSC delivered the maximum energy density of 44.4 Wh kg^−1^ at a power density of 440 W kg^−1^; moreover, with further increase to 5255 W kg^−1^, an energy density of 26.1 Wh kg^−1^ still could be attained, revealing its outstanding energy storage performance. Compared with traditional supercapacitors, our assembled device exhibits prominent advantage in energy density; meanwhile, its desired power density is also superior to that of several kinds of batteries. On account of above-mentioned findings, the remarkable electrochemical performances of Ni-MOF/C-CNTs could be mainly attributed to their ultrathin 2D sheet-like nanostructure. Particularly, the C-CNTs provided fast axial electron transport and induced the extension of Ni-MOF nanosheets, thus forming a highly porous and continuous structure with large specific surface area to accelerate the penetration of OH^−^, as well as abundant active sites to enhance the electrochemical reaction kinetics.

## Conclusions

In summary, an in situ induced growth strategy has been employed to synthesize ultrathin 2D C-CNTs interpenetrated nickel MOFs (Ni-MOF/C-CNTs) nanosheets. The incorporated C-CNTs not only successfully adjust the layer thickness of Ni-MOF, but also improve the charge transfer within hybrid nanostructure. By judicious design, the integrated MOFs hybrids with ultrathin nanosheets were endowed with abundant electroactive sites, thus significantly advancing the electrochemical performances. As a result, the Ni-MOF/C-CNTs40 exhibits excellent specific capacity of 680 C g^−1^ at current density of 1 A g^−1^ and the 68% capacity retention at 10 A g^−1^. Moreover, the Ni-MOF/C-CNTs40//AC hybrid device delivered good energy storage capacity with a maximum energy density of 44.4 Wh kg^−1^ at a power density of 440 W kg^−1^, and a desired cycling stability. This facile, controllable strategy for the development of ultrathin 2D MOF can also be extended to other MOF-based functional materials and metal hydroxides for specific applications.

## Electronic supplementary material

Below is the link to the electronic supplementary material.

Supplementary material 1 (PDF 1135 kb)
