# Supplementary material for: Ultrathin 2D Metal–Organic Framework Nanosheets In situ Interpenetrated by Functional CNTs for Hybrid Energy Storage Device
Source: Nanomicro Lett. 2020 Feb 4;12:46. doi: 10.1007/s40820-020-0382-x (PMC7770780; doi:10.1007/s40820-020-0382-x)
Supplement: Supplementary file 1 — Supplementary material 1 (PDF 1135 kb) [file 40820_2020_382_MOESM1_ESM.pdf]

Supporting Information for

Ultrathin 2D Metal Organic Framework Nanosheets In-situ Interpenetrated by Functional CNTs for Hybrid Energy Storage Device

Feitian Ran[1], Xueqing Xu[1], Duo Pan[2], Yuyan Liu[1], Yongping Bai[1], Lu Shao[1, *]

[1]MIIT Key Laboratory of Critical Materials Technology for New Energy Conversion and Storage, State Key Laboratory of Urban Water Resource and Environment (SKLUWRE), School of Chemistry and Chemical Engineering, Harbin Institute of Technology, Harbin 150001, People's Republic of China

[2]Key Laboratory of Materials Processing and Mold (Zhengzhou University), Ministry of Education; National Engineering Research Center for Advanced Polymer Processing Technology, Zhengzhou University, Zhengzhou 450002, People's Republic of China

*Corresponding author. E-mail: shaolu@hit.edu.cn (Lu Shao)

S1 Experimental Section

S1.1 Materials

Nickel nitrate hexahydrate ($Ni(NO_3)_2 \cdot 6H_2O$), 1,3,5-benzenetricarboxylic acid (H_3BTC), and potassium hydroxide (KOH) were purchased from Aladdin Chemicals. N, N-dimethylformamide (DMF) was obtained from Xilong Chemical Co., Ltd (China). Carboxylated multi-walled carbon nanotubes (CNTs) were purchased from Nanjing XFNANO Materials Tech Co., Ltd (China). Commercial activated carbon (AC, YEC-8, FUZHOU YIHUAN CARBON., LTD). NKK separator (MPF30AC-100, Nippon Kodoshi Corporation, Kochi, Japan). All chemical reagents were analytical purity and without further purification.

S1.2 Characterization

The microstructures and morphologies of samples were observed *via* field-emission scanning electron microscopy (FE-SEM, MERLIN Compact, Carl Zeiss, Germany) and transmission electron microscopy (TEM, Tecnai G2 F30, Netherlands). Before test, the appropriate amounts of obtained samples were uniformly dispersed into the ethanol under ultrasonic, and then dropped on the cleaned silicon slice using glass capillary. After dried, the prepared silicon slice was pasted on the aluminum substrate using conductive tape to perform the SEM and EDS characterizations. The crystal structures of samples were identified by powder X-ray diffraction (XRD, Bruker D8 Advance X-ray diffractometer, Cu Kα radiation, λ = 1.540598 Å). The X-ray photoelectron spectroscopy (XPS) measurements were conducted on an AXIS ULTRA DLD spectrometer (SHIMADZU, Japan) to analyze surface elemental composition and

valence state. Nitrogen adsorption/desorption isotherms were conducted on BELSORP-mini-II (BEL Japan) adsorption analyzer. The specific surface area was calculated using the Brunauer-Emmett-Teller (BET) method. The pore size distribution was calculated from the corresponding adsorption branch of N_2 isotherm by BJH method for mesopores. Fourier transform infrared spectroscopy (FTIR) was conducted on a Spectrum One instrument (Perkin Elmer, USA).

S1.3 Calculation Formulas

The specific capacity (C g^{-1}) was calculated according to the following equations [S1, S2]:

$$C = \frac{I\Delta t}{m}$$

Where I (A) is the discharge current, Δt (s) is the discharge time, m (g) is the mass loading of the active material.

The charge storage of the positive and negative electrodes was balanced by controlling the loading mass ratio of positive electrode material and negative electrode material using the following equation [S3].

$$Q^{+} = Q^{-}$$

$$Q = mC$$

$$\frac{m_{+}}{m_{-}} = \frac{C_{-}}{C_{+}}$$

Where Q is the charge stored by each electrode, m (g) is the mass of the active material, and C (C g^{-1}) is the specific capacity.

The specific capacitances C_s (F g^{-1}) of HSC device were calculated from the GCD data according to following equation [S4, S5]:

$$C_s = \frac{2i_m \int V dt}{V^2 \Big|_{V_i}^{V_f}}$$

Where $\int V dt$ is the integral area of the discharge curve, and the V (V) is the potential with initial and final values of V_i and V_f, respectively. $i_m = I/m$ (A g^{-1}) is the current density, where I is the current and m is the mass of active materials.

The energy densities E (Wh kg^{-1}) and power densities P (W kg^{-1}) of HSC device were calculated as follows [S2, S5, S6]:

$$E = \frac{i_m \int V dt}{3.6}$$

$$P = \frac{E}{t} \times 3600$$

Where t (s) is the discharge time.

Table S1 Production yield of Ni-MOF and a series of Ni-MOF/C-CNTs

Sample	Raw material (g)			Product (g)	Yield
	$Ni(NO_3)_2·6H_2O$	H_3BTC	C-CNTs		
Ni-MOF	0.872	0.105	0	0.456	46.7%
Ni-MOF/C-CNTs20	0.872	0.105	0.020	0.475	47.6%
Ni-MOF/C-CNTs40	0.872	0.105	0.040	0.519	48.5%
Ni-MOF/C-CNTs60	0.872	0.105	0.060	0.548	52.8%

S2 Supplementary Figures and Tables

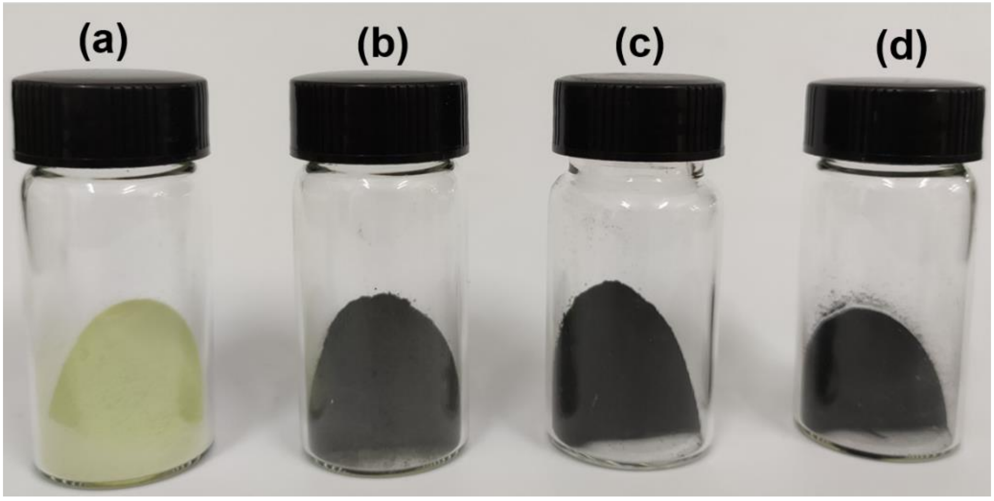

Fig. S1 Electronic photos of as-obtained **a** Ni-MOF, **b** Ni-MOF/C-CNTs20, **c** Ni-MOF/C-CNTs40 and **d** Ni-MOF/C-CNTs60.

As observed, the pure Ni-MOF present light green color; after incorporated C-CNTs, the obtained Ni-MOF/C-CNTs become black color. Regarding to volume samples, it can be observed that the appropriate amount of C-CNTs has result in slightly increment of volume; however, with increasing the C-CNTs amount, a volume shrink was observed, which can be attributed to the agglomeration caused by sharp increase of nucleation sites.

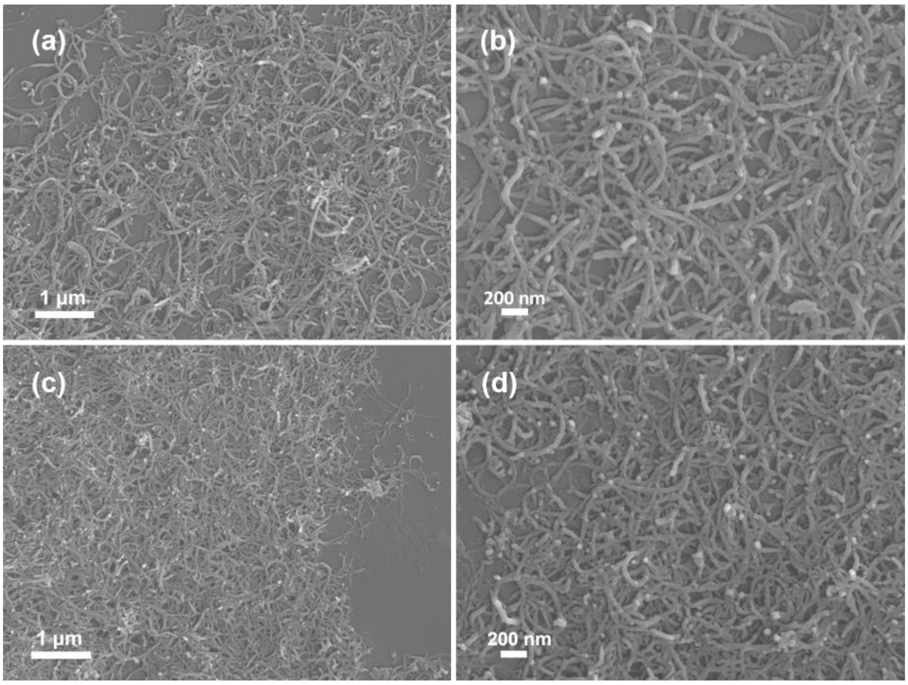

Fig. S2 SEM images of **a**, **b** C-CNTs and **c, d** CNTs

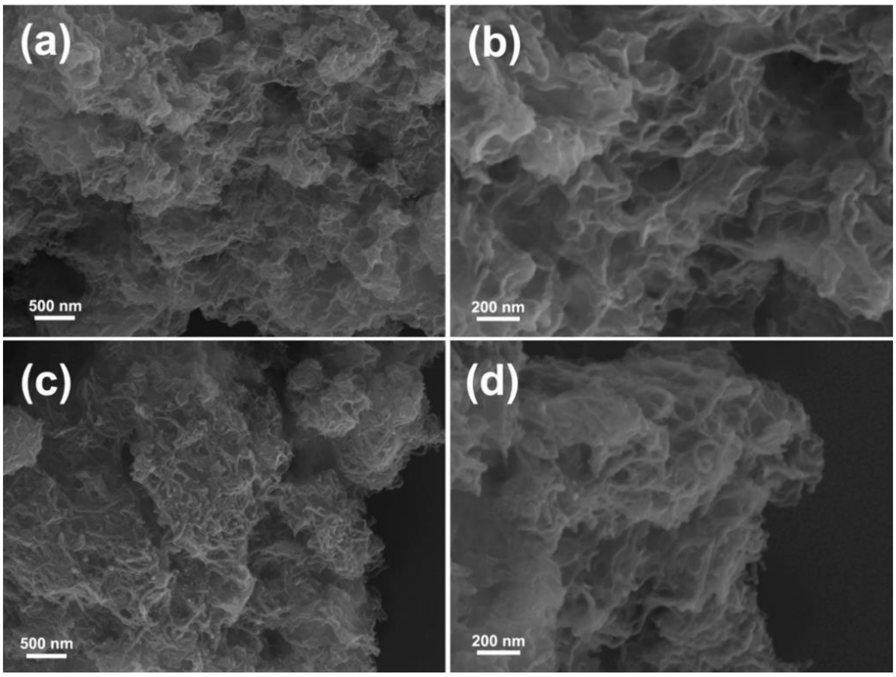

Fig. S3 SEM images of as-synthesized **a**, **b** Ni-MOFs20 and **c, d** Ni-MOF/C-CNTs60

As shown in **Fig. S3a, b,** the small amount of C-CNTs have no obvious influence on morphology evolution of Ni-MOF, the stacked nanosheets can still be observed. However, when the content of C-CNTs increase continuously, an agglomeration tendency have appeared for Ni-MOF/C-CNTs60 (**Fig. S3c, d**). These observation reveal that the moderate amounts of C-CNTs is very important for constructing the ultrathin 2D well-interconnected nanosheets.

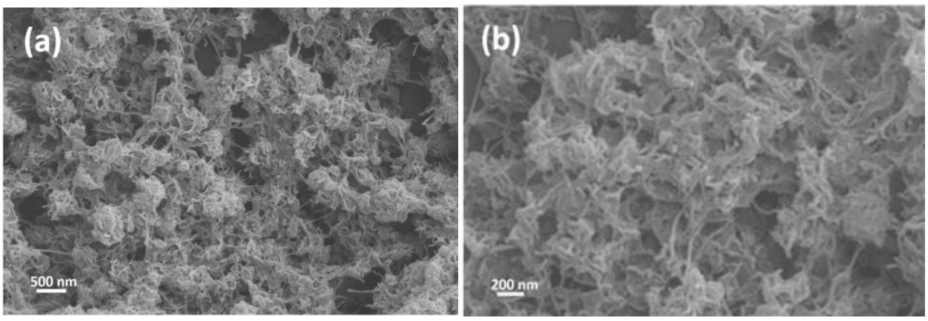

Fig. S4 SEM images of Ni-MOF/CNTs40

As shown in **Fig. S4**, different from the layer-by-layer stacking structures of pristine Ni-MOF, the Ni-MOF/CNTs40 appear loose nanostructure, indicating the CNTs has the effect on crystallization of Ni-MOF. However, we can observe plentiful CNTs on the surface of Ni-MOF/CNTs40 hybrid instead of embedding in Ni-MOF, which is totally different from the well-interconnected ultrathin nanosheets of Ni-MOF/C-CNTs40. This result indicates that the C/O groups of C-CNTs play a significant role in the nucleate and growth process.

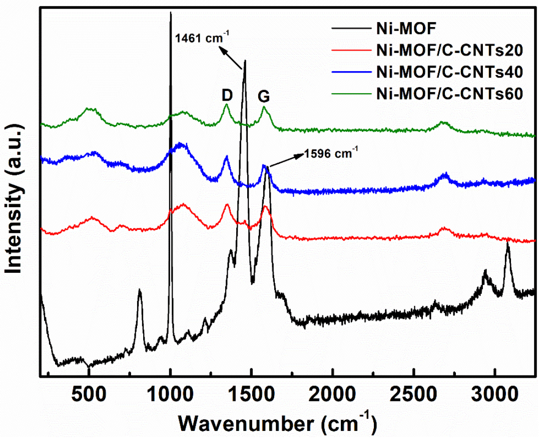

Fig. S5 Raman spectra of Ni-MOF and a series of Ni-MOF/C-CNTs nanohybrids

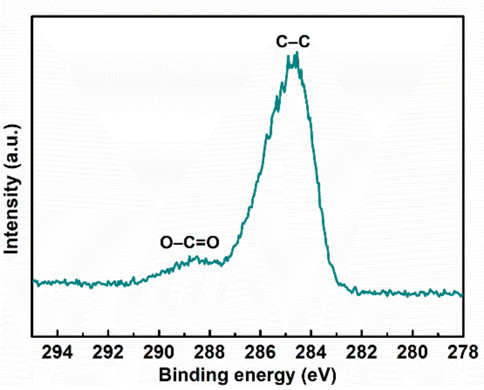

Fig. S6 High-resolution XPS spectrum for C 1s of Ni-MOF/C-CNTs40

Two major peaks at 284.5 and 288.5 eV correspond to phenyl carbons (C=C) and carboxylate carbons (O–C=O).

Table S2 Comparison of electrochemical performance for different Ni/Co MOFs based electrode materials in alkaline electrolyte

Electrode materials	Potential window (V)	Specific capacitance (capacity)	Capacitance retention	References
Pillared Ni-MOFs	0-0.45 (*vs.* SCE)	552 F g^{-1} (1 A g^{-1})	71.6 % (50 A g^{-1})	[S7]
CoNi-MOF	0-0.45 (*vs.* SCE)	1104 F g^{-1} (1 A g^{-1})	52 % (32 A g^{-1})	[S8]
2D Ni-MOF	0-0.35 (*vs.* SCE)	1127 F g^{-1} (0.5 A g^{-1})	59.3 % (10 A g^{-1})	[S9]
Ni-MOF	0-0.45 (*vs.*Hg/HgO)	1021 F g^{-1} (0.7 A g^{-1})	80.6 % (7 A g^{-1})	[S10]
Zn-doped Ni-MOF	0-0.35 (*vs.* SCE)	1620 F g^{-1} (0.25 A g^{-1})	57.3 % (10 A g^{-1})	[S11]
Ni/Co-MOF	0-0.55 (*vs.* Ag/AgCl)	530.4 F g^{-1} (0.5 A g^{-1})	/	[S12]
Ni-Tp/PANI	0-0.55 (*vs.* Hg/HgO)	938.8 F g^{-1} (1.8 A g^{-1})	19.4 % (9 A g^{-1})	[S13]
GM-LEG@Ni-MOF	0-0.35 (*vs.* SCE)	987.6 F g^{-1} (0.5 A g^{-1})	69.4% (10 A g^{-1})	[S14]
Zn-doped Ni-MOF	0-0.45 (*vs.* SCE)	237.4 mAh g^{-1} (1 A g^{-1})	51.5% (10 A g^{-1})	[S15]
2D Ni-MOF/C-CNTs	0-0.54 (*vs.* Hg/HgO)	1259 F g^{-1}/680 C g^{1}(1 A g^{-1})	63 % (10 A g^{-1})	This work

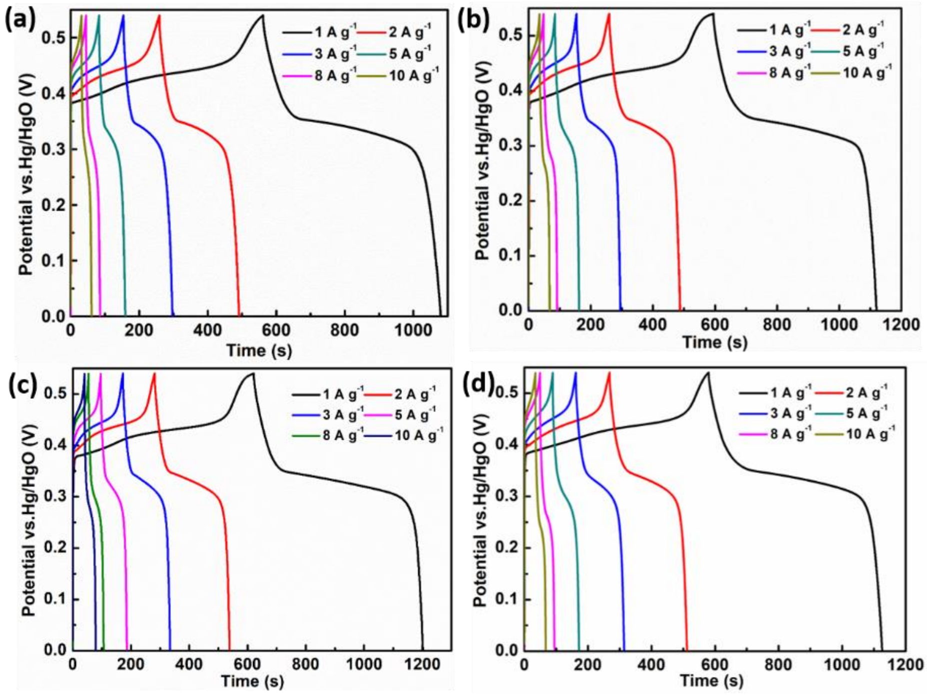

Fig. S7 Galvanostatic charge-discharge curves of **a** Ni-MOF, **b** Ni-MOF/C-CNTs20, **c** Ni-MOF/C-CNTs60 and **d** Ni-MOF/CNTs40

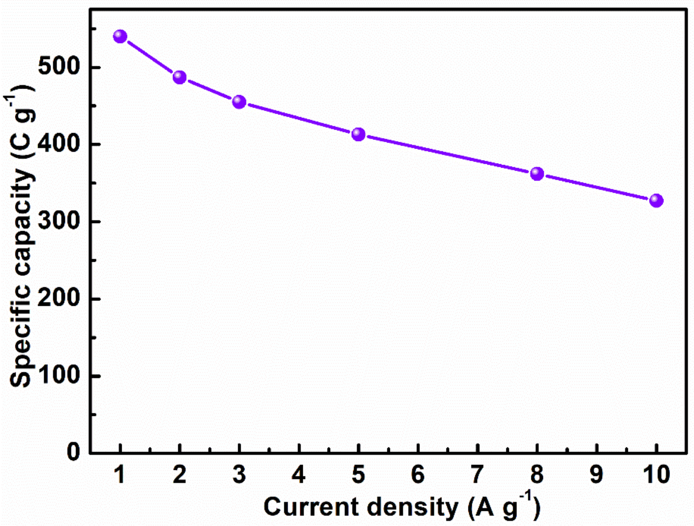

Fig. S8 Specific capacity at various current densities for Ni-MOF/CNTs40

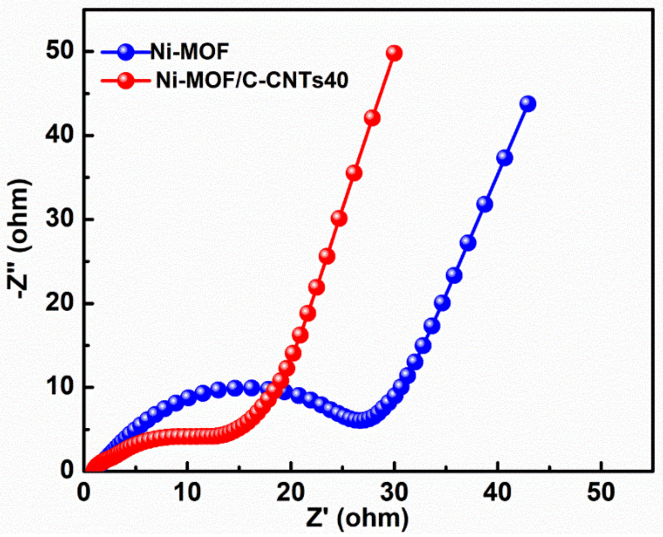

Fig. S9 Comparison of Nyquist plots for Ni-MOFs Ni-MOF/C-CNTs40

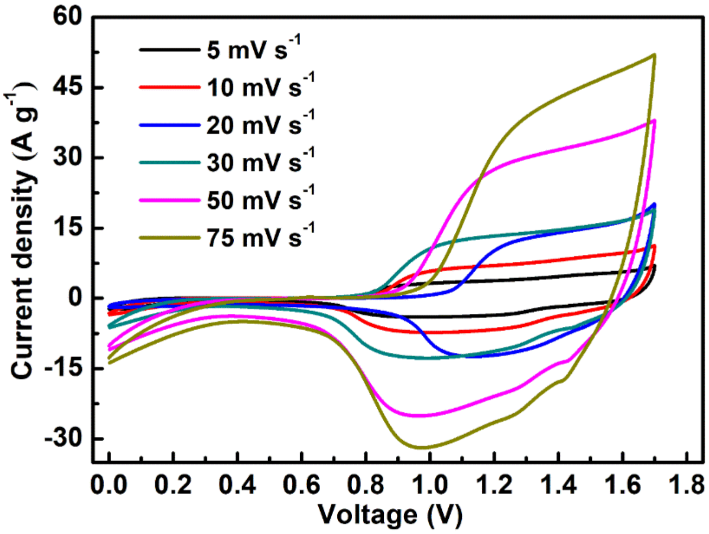

Fig. S10 CV curves of the HSC device in various scan rates

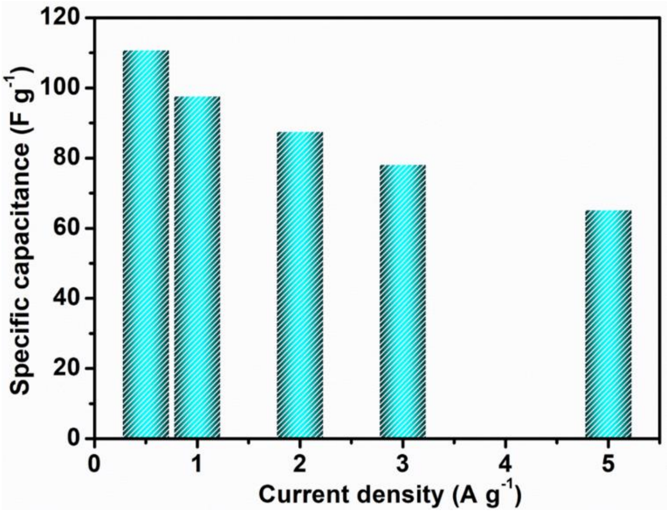

Fig. S11 Specific capacitances with respect to the current densities of HSC device

Supplementary References

[S1]X. Liu, W. Zang, C. Guan, L. Zhang, Y. Qian et al., Ni-doped cobalt-cobalt nitride heterostructure arrays for high-power supercapacitors. ACS Energy Lett. **3**, 2462-2469 (2018). https://doi.org/10.1021/acsenergylett.8b01393

[S2]B. Zhao, L. Zhang, Q. Zhang, D. Chen, Y. Cheng et al., Rational design of nickel hydroxide-based nanocrystals on graphene for ultrafast energy storage. Adv. Energy Mater. **8**, 1702247 (2018). https://doi.org/10.1002/aenm.201702247

[S3]P. Sun, W. He, H. Yang, R. Cao, J. Yin, C. Wang, X. Xu, Hedgehog-inspired nanostructures for hydrogel-based all-solid-state hybrid supercapacitors with excellent flexibility and electrochemical performance. Nanoscale **10**, 19004-19013 (2018). https://doi.org/10.1039/c8nr04919j

[S4]P. Yang, Z. Wu, Y. Jiang, Z. Pan, W. Tian, L. Jiang, L. Hu, Fractal $(Ni_xCo_{1-x})_9Se_8$ nanodendrite arrays with highly exposed (011¯) surface for wearable, all-solid-state supercapacitor. Adv. Energy Mater. **8**, 1801392 (2018). https://doi.org/10.1002/aenm.201801392

[S5]J. Balamurugan, C. Li, V. Aravindan, N.H. Kim, J.H. Lee, Hierarchical Ni-Mo-S and Ni-Fe-S nanosheets with ultrahigh energy density for flexible all solid-state supercapacitors. Adv. Funct Mater **28**, 1803287 (2018). https://doi.org/10.1002/adfm.201803287

[S6]B. Zhao, D. Chen, X. Xiong, B. Song, R. Hu et al., A high-energy, long cycle-life hybrid supercapacitor based on graphene composite electrodes. Energy Storage Mater. **7**, 32-39 (2017). https://doi.org/10.1016/j.ensm.2016.11.010

[S7]C. Qu, Y. Jiao, B. Zhao, D. Chen, R. Zou, K.S. Walton, M. Liu, Nickel-based pillared mofs for high-performance supercapacitors: design, synthesis and stability study. Nano Energy **26**, 66-73 (2016).

https://doi.org/10.1016/j.nanoen.2016.04.003

[S8]T. Deng, Y. Lu, W. Zhang, M. Sui, X. Shi, D. Wang, W. Zheng, Inverted design for high-performance supercapacitor via $Co(OH)_2$-derived highly oriented MOF electrodes. Adv. Energy Mater. **8**, 1702294 (2018). https://doi.org/10.1002/aenm.201702294

[S9]J. Yang, P. Xiong, C. Zheng, H. Qiu, M. Wei, Metal–organic frameworks: a new promising class of materials for a high performance supercapacitor electrode. J. Mater. Chem. A **2**, 16640-16644 (2014). https://doi.org/10.1039/c4ta04140b

[S10] Y. Yan, P. Gu, S. Zheng, M. Zheng, H. Pang, H. Xue, Facile synthesis of an accordion-like Ni-MOF superstructure for high-performance flexible supercapacitors. J. Mater. Chem. A **4**, 19078-19085 (2016). https://doi.org/10.1039/c6ta08331e

[S11] J. Yang, C. Zheng, P. Xiong, Y. Li, M. Wei, Zn-doped Ni-MOF material with a high supercapacitive performance. J. Mater. Chem. A **2**, 19005-19010 (2014). https://doi.org/10.1039/c4ta04346d

[S12] H. Xia, J. Zhang, Z. Yang, S. Guo, S. Guo, Q. Xu, 2D MOF nanoflake-assembled spherical microstructures for enhanced supercapacitor and electrocatalysis performances. Nano-Micro Lett. **9**, 43 (2017). https://doi.org/10.1007/s40820-017-0144-6

[S13] Q. Chen, S. Lei, P. Deng, X. Ou, L. Chen, W. Wang, Y. Xiao, B. Cheng, Direct growth of nickel terephthalate on Ni foam with large mass-loading for high-performance supercapacitors. J. Mater. Chem. A **5**, 19323-19332 (2017). https://doi.org/10.1039/c7ta05373h

[S14] Y. Xiao, W. Wei, M. Zhang, S. Jiao, Y. Shi, S. Ding, Facile surface properties engineering of high-quality graphene: toward advanced Ni-MOF heterostructures for high-performance supercapacitor electrode. ACS Appl. Energy Mater. **2**, 2169-2177 (2019). https://doi.org/10.1021/acsaem.8b02201

[S15] Y. Chen, D. Ni, X. Yang, C. Liu, J. Yin, K. Cai, Microwave-assisted synthesis of honeycomblike hierarchical spherical Zn-doped Ni-MOF as a high-performance battery-type supercapacitor electrode material. Electrochim. Acta **278**, 114-123 (2018). https://doi.org/10.1016/j.electacta.2018.05.024
